# Plant Disease Detection and Classification by Deep Learning

**DOI:** 10.3390/plants8110468

**Published:** 2019-10-31

**Authors:** Muhammad Hammad Saleem, Johan Potgieter, Khalid Mahmood Arif

**Affiliations:** 1 Department of Mechanical and Electrical Engineering, School of Food and Advanced Technology, Massey University, Auckland 0632, New Zealand; h.saleem@massey.ac.nz; 2 Massey Agritech Partnership Research Centre, School of Food and Advanced Technology, Massey University, Palmerston North 4442, New Zealand; j.potgieter@massey.ac.nz

**Keywords:** plant disease, deep learning, convolutional neural networks (CNN)

## Abstract

Plant diseases affect the growth of their respective species, therefore their early identification is very important. Many Machine Learning (ML) models have been employed for the detection and classification of plant diseases but, after the advancements in a subset of ML, that is, Deep Learning (DL), this area of research appears to have great potential in terms of increased accuracy. Many developed/modified DL architectures are implemented along with several visualization techniques to detect and classify the symptoms of plant diseases. Moreover, several performance metrics are used for the evaluation of these architectures/techniques. This review provides a comprehensive explanation of DL models used to visualize various plant diseases. In addition, some research gaps are identified from which to obtain greater transparency for detecting diseases in plants, even before their symptoms appear clearly.

## 1. Introduction

**Figure 1:**
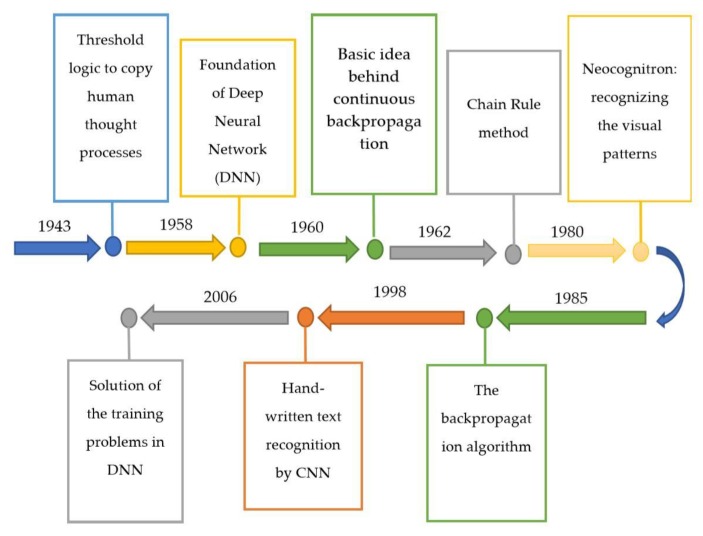
Summary of the evolution of deep learning from 1943–2006.

**Figure 2:**
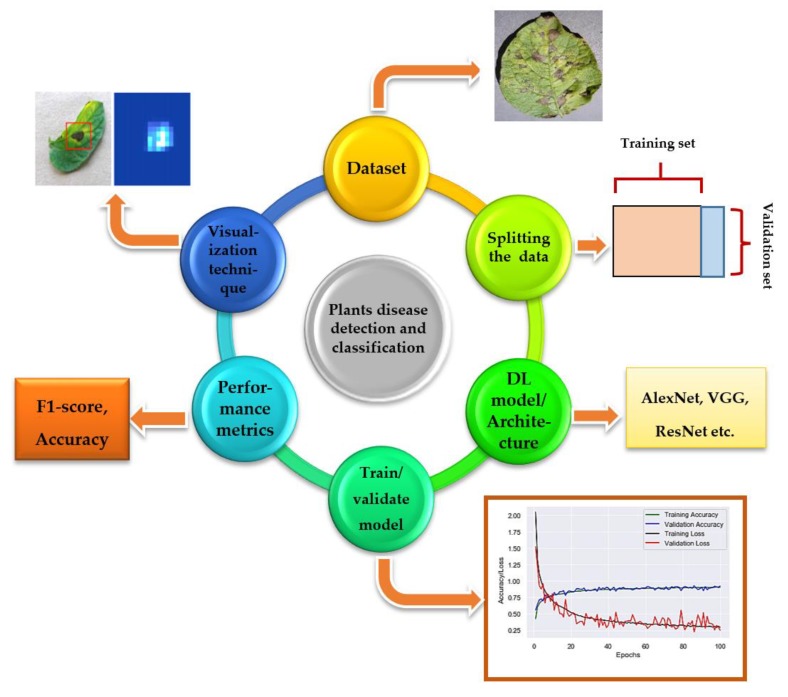
Flow diagram of DL implementation: First, the dataset is collected [25] then split into two parts, normally into 80% of training and 20% of validation set. After that, DL models are trained from scratch or by using transfer learning technique, and their training/validation plots are obtained to indicate the significance of the models. Then, performance metrics are used for the classification of images (type of particular plant disease), and finally, visualization techniques/mappings [55] are used to detect/localize/classify the images.

The Deep Learning (DL) approach is a subcategory of Machine Learning (ML), introduced in 1943 [1] when threshold logic was introduced to build a computer model closely resembling the biological pathways of humans. This field of research is still evolving; its evolution can be divided into two time periods-from 1943–2006 and from 2012–until now. During the first phase, several developments like backpropagation [2,3], chain rule [4], Neocognitron [5], hand written text recognition (LeNET architecture) [6], and resolving the training problem [7,8] were observed (as shown in Figure 1). However, in the second phase, state-of-the-art algorithms/architectures were developed for many applications including self-driving cars [9,10,11], healthcare sector [12,13,14], text recognition [6,15,16,17], earthquake predictions [18,19,20], marketing [21], finance [22,23], and image recognition [24,25,26,27,28,29]. Among those architectures, AlexNet [30] is considered to be a breakthrough in the field of DL as it won the ImageNet challenge for object recognition known as ImageNet Large Scale Visual Recognition Challenge (ILSVRC) in the year 2012. Soon after, several architectures were introduced to overcome the loopholes observed previously. For the evaluation of these algorithms/architectures, various performance metrics were used. Among these metrics, top-1%/top-5% error [24,26,30,31], precision and recall [25,32,33,34], F1 score [32,35], training/validation accuracy and loss [34,36], classification accuracy (CA) [37,38,39,40,41] are the most popular. For the implementation of DL models, several steps are required, from the collection of datasets to visualization mappings are explained in Figure 2.

When DL architectures started to evolve with the passage of time, researchers applied them to image recognition and classification. These architectures have also been implemented for different agricultural applications. For example, in [42], classification of leaves was performed by using author-modified CNN and Random Forest (RF) classifier among 32 species in which the performance was evaluated through CA at 97.3%. On the other hand, it was not as efficient at detecting occluded objects [43]. Leaf and fruit counting were also performed by deep CNN in [44,45] and [46] respectively. For classification of crop type, [47] used author-modified CNN, [36] applied VGG 16, [34] implemented three unit LSTM, and [33] used CNN and RGB histogram technique. [47] used CA, [36] used CA and Intersection over Union (IoU), [34] used CA and F1, and [33] used F1-score as a performance metric. Among them, [33,47] did not provide training/validation accuracy and loss. Moreover, recognition of different plants has been done by the DL approach in [48,49,50]. [48,50] employed user-modified CNN while [49] used AlexNet architecture. All were evaluated on the basis of CA. [49] outperformed the other two in terms of CA. Similarly, crop/weed discrimination was performed in [51,52], in which the author proposed CNN be used, and two datasets were utilized for the evaluation of the model. [51] evaluated precision and recall; however, [52] obtained CA for the validation of the proposed models respectively. The identification of plants by the DL approach was studied and achieved a success rate of 91.78% [53]. On top of that, DL approaches are also used for critical tasks like plant disease detection and classification, which is the main focus of this review. There are some research papers previously presented to summarize the research based on agriculture (including plant disease recognition) by DL [43,54], but they lacked some of the recent developments in terms of visualization techniques implemented along with the DL and modified/cascaded version of famous DL models, which were used for plant disease identification. Moreover, this review also provides the research gaps in order to get a clearer/more transparent vision of symptoms observed due to diseases in the plants. 

The remaining part of the paper is comprised of Section 2, describing the famous and new/modified DL architectures along with visualization mapping/techniques used for plant disease detection; Section 3, elaborating upon the Hyperspectral Imaging with DL models; and finally, Section 4, concluding the review and providing future recommendations for achieving more advancements in the visualization, detection, and classification of plants’ diseases. 

## 2. Plant Disease Detection by Well-Known DL Architectures

**Figure 3:**
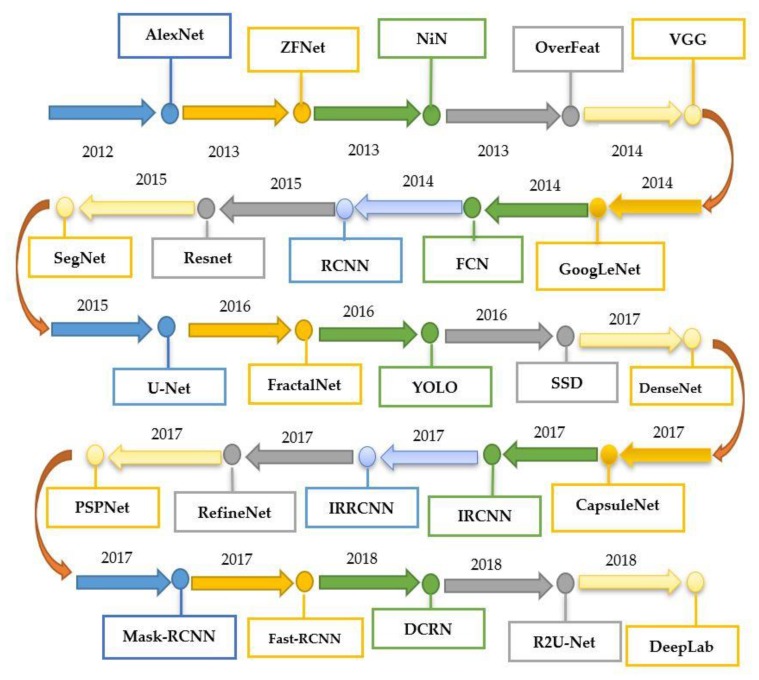
Summary of the evolution of various deep learning models from 2012 until now.

Many state-of-the-art DL models/architectures evolved after the introduction of AlexNet [30] (as shown in Figure 3 and Table 1) for image detection, segmentation, and classification. This section presents the researches done by using famous DL architectures for the identification and classification of plants’ diseases. Moreover, there are some related works in which new visualization techniques and modified/improved versions of DL architectures were introduced to achieve better results. Among all of them, the PlantVillage dataset has been used widely as it contains 54,306 images of 14 different crops having 26 plant diseases [25]. Moreover, they used several performance metrics to evaluate the selected DL models, which are described as below.

### 2.1. Implementation of DL Models

#### 2.1.1. Without Visualization Technique

In [56], CNN was used for the classification of diseases in maize plants and histogram techniques to show the significance of the model. In [57], basic CNN architectures like AlexNet, GoogLeNet and ResNet were implemented for identifying the tomato leaf diseases. Training/validation accuracy were plotted to show the performance of the model; ResNet was considered as the best among all the CNN architectures. In order to detect the diseases in banana leaf, LeNet architecture was implemented and CA, F1-score were used for the evaluation of the model in Color and Gray Scale modes [32]. Five CNN architectures were used in [58], namely, AlexNet, AlexNetOWTbn, GoogLeNet, Overfeat, and VGG architectures in which VGG outclassed all the other models. In [35], eight different plant diseases were recognized by three classifiers, Support Vector Machines (SVM), Extreme Learning Machine (ELM), and K-Nearest Neighbor (KNN)), used with the state-of-the-art DL models like GoogLeNet, ResNet-50, ResNet-101, Inception-v3, InceptionResNetv2, and SqueezeNet. A comparison was made between those models, and ResNet-50 with SVM classifier got the best results in terms of performance metrics like sensitivity, specificity, and F1-score. According to [59], a new DL model—Inception-v3—was used for the detection of cassava disease. In [60], plant diseases in cucumber were classified by the two basic versions of CNN and got the highest accuracy, equal to 0.823. The traditional plant disease recognition and classification method was replaced by Super-Resolution Convolutional Neural Network (SRCNN) in [61]. For the classification of tomato plant disease, AlexNet and SqueezeNet v1.1 models were used in which AlexNet was found to be the better DL model in terms of accuracy [62]. A comparative analysis was presented in [63] to select the best DL architecture for detection of plant diseases. Moreover in [64], six tomato plant diseases were classified by using AlexNet and VGG-16 DL architectures, and a detailed comparison was provided with the help of classification accuracy. In the above approaches, no visualization technique was applied to spot the symptoms of diseases in the plants. 

#### 2.1.2. With Visualization Techniques

The following approaches employed DL models/architectures and also visualization techniques which were introduced for a clearer understanding of plants’ diseases. For example, [55] introduced the saliency map for visualizing the symptoms of plant disease; [27] identified 13 different types of plant disease with the help of CaffeNet CNN architecture, and achieved CA equal to 96.30%, which was better than the previous approach like SVM. Moreover, several filters were used to indicate the disease spots. Similarly, [25] used AlexNet and GoogLeNet CNN architectures by using the publicly available PlantVillage dataset. The performance was evaluated by means of precision (P), recall (R), F1 score, and overall accuracy. The uniqueness of this paper was the implication of three scenarios (color, grayscale, and segmented) for evaluating the performance metrics and comparison of the two famous CNN architectures. It was concluded that GoogLeNet outperformed AlexNet. Moreover, visualization activation in the first layers clearly showed the spots of diseases. In [65], a modified LeNet model was used to detect olive plant diseases. The segmentation and edges maps were used to spot the diseases in the plants. Detection of four cucumber diseases was done in [66] and accuracy was compared with Random Forest, Support Vector Machines, and AlexNet models. Moreover, the image segmentation method was used to view the symptoms of diseases in the plants. A new DL model was introduced in [67] named teacher/student network and proposed a novel visualization method to identify the spots of plant diseases. DL models with some detectors were implemented in [68], in which the diseases in plants were marked along with their prediction percentage. Three detectors, named Faster-RCNN, RFCN and SSD, were used with the famous architectures like AlexNet, GoogLeNet, VGG, ZFNet, ResNet-50, ResNet-101 and ResNetXt-101 for a comparative study which outlined the best among all the selected architectures. It was concluded that ResNet-50 with the detector R-FCN gave the best results. Furthermore, a kind of bounding box was drawn to identify the particular type of disease in the plants. In [69], a banana leaf disease and pest detection was performed by using three CNN models (ResNet-50, Inception-V2 and MobileNet-V1) with Faster-RCNN and SSD detectors. According to [70], different combinations of CNN were used and presented heat maps as input to the diseased plants’ images and provided the probability related to the occurrence of a particular type of disease. Moreover, ROC curve evaluates the performance of the model. Furthermore, feature maps for rice disease were also included in the paper. LeNet model was used in [71] to detect and classify diseases in the soybean plant. In [72], a comparison between AlexNet and GoogLeNet architectures for tomato plant diseases was done, in which GoogLeNet performed better than the AlexNet; also, it proposed occlusion techniques to recognize the regions of diseases. The VGG-FCN and VGG-CNN models were implemented in [73], for the detection of wheat plant diseases and visualization of features in each block. In [74], VGG-CNN model was used for the detection of Fusarium wilt in radish and K-means clustering method was used to show the marks of diseases. A semantic segmentation approach by CNN was proposed in [75] to detect the disease in cucumber. In [76], an approach based on the individual symptoms/spots of diseases in the plants was introduced by using a DL model for detecting plant diseases. A Deep CNN framework was developed for identification, classification, and quantification of eight soybean stresses in [77]. In [78], rice plant diseases were identified by CNN, and feature maps were obtained to identify the patches of diseases. A deep residual neural network was extended in [79] for the development of a mobile application in which a clear identification of diseases in plants was done by the hot spot. An algorithm based on the hot spot technique was also used in [80], in which those spots were extracted by modification in the segmented image to attain color constancy. Furthermore, each obtained hot-spot was described by two descriptors, one was used to evaluate the color information of the disease and other was used to identify the texture of the hot-spots. The cucumber plant diseases were identified in [81] by using the dilation convolutional neural network. A state-of-the-art visualization technique was proposed in [82] by correlation coefficient and DL models like AlexNet and VGG-16 architectures. In [83], color space and various vegetation indices combined with CNN model (LeNet) to detect the diseases in grapes. To summarize, Table 2 outlines some of the visualization mapping/techniques. 

**Figure 4:**
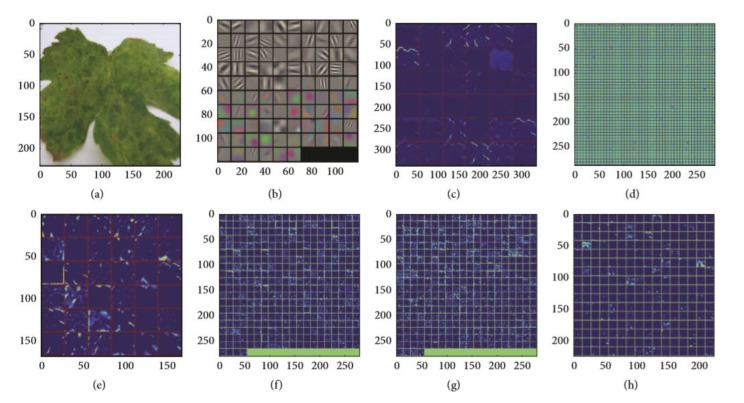
Feature maps after the application of convolution to an image: (**a**) real image, (**b**) first convolutional layer filter, (**c**) rectified output from first layer, (**d**) second convolutional layer filter, (**e**) output from second layer, (**f**) output of third layer, (**g**) output of fourth layer, (**h**) output of fifth layer [27].

**Figure 5:**
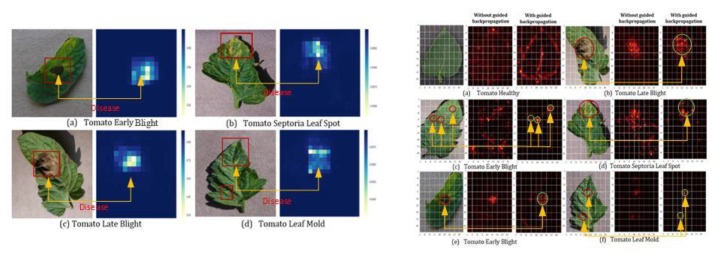
Tomato plant disease detection by heat map: on left hand side (**a**) tomato early blight, (**b**) tomato septoria leaf spot, (**c**) tomato late blight and (**d**) tomato leaf mold) and saliency map; on right hand side (**a**) tomato healthy, (**b**) tomato late blight, (**c**) tomato early blight, (**d**) tomato septoria leaf spot, (**e**) tomato early blight, (**f**) tomato leaf mold) [55].

**Figure 6:**
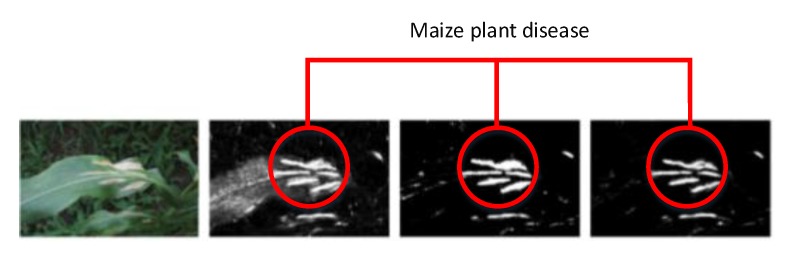
Detection of maize disease (indicated by red circles) by heat map [70].

**Figure 7:**
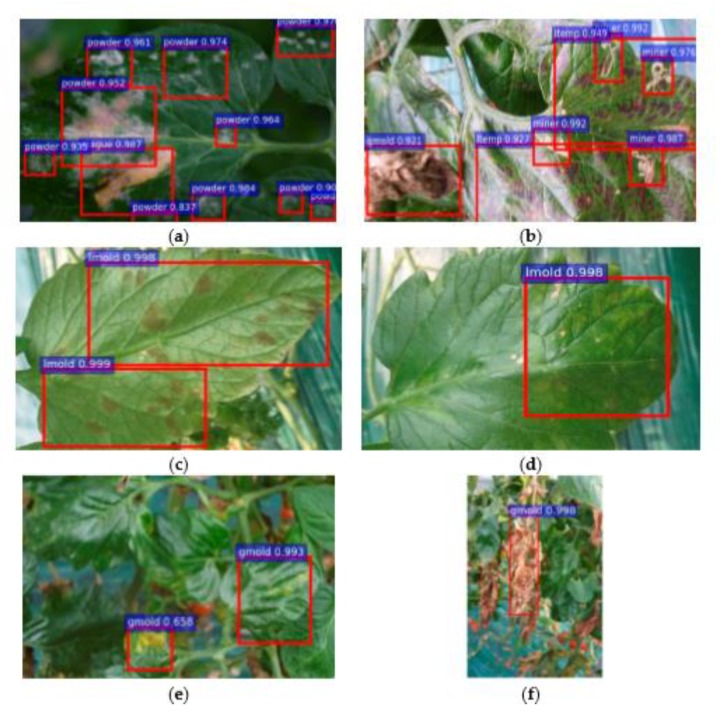
Bounding box indicates the type of diseases along with the probability of their occurrence [68]. A bounding box technique was used in Figure 7 in which (**a**) represents the one type of disease along with its rate of occurrence, (**b**) indicates three types of plant disease (miner, temperature, and gray mold) in a single image, (**c**,**d**) shows one class of disease but contains different patterns on the front and back side of the image, (**e**,**f**) displays different patterns of gray mold in the starting and end stages [68].

**Figure 8:**
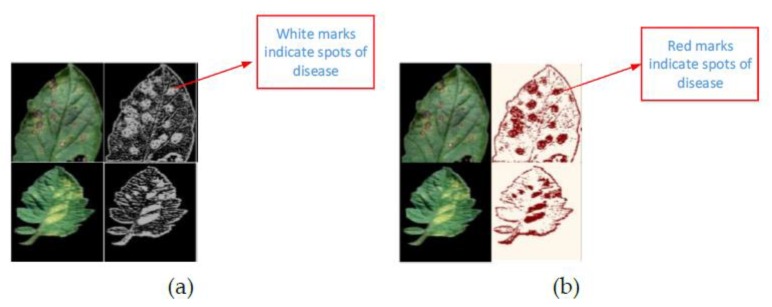
(**a**) Teacher/student architecture approach; (**b**) segmentation using a binary threshold algorithm [67].

**Figure 9:**
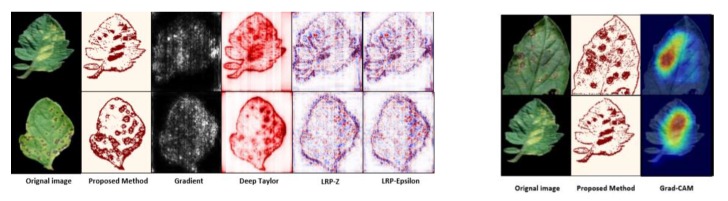
Comparison of Teacher/student approach visualization map with the previous approaches [67].

**Figure 10:**
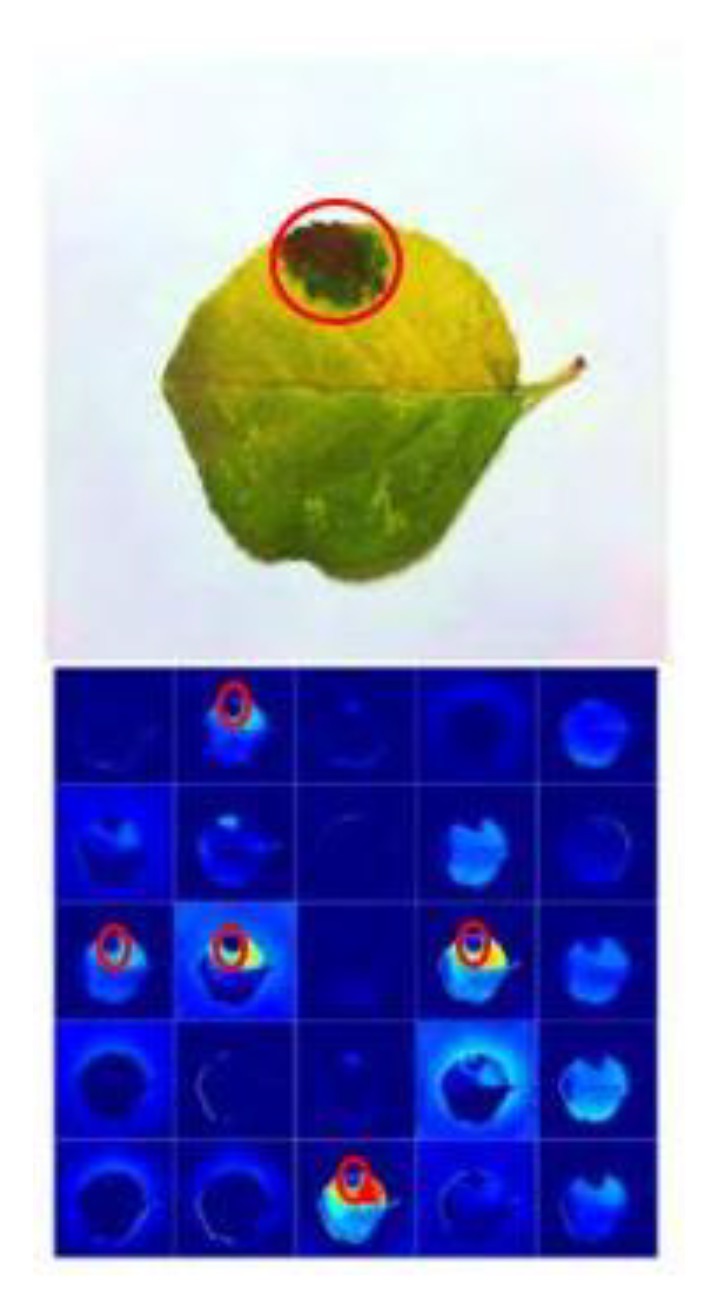
Activation visualization for detection of apple plant disease to show the significance of a VGG-Inception model (the plant disease is indicated by the red circle) [85].

**Figure 11:**
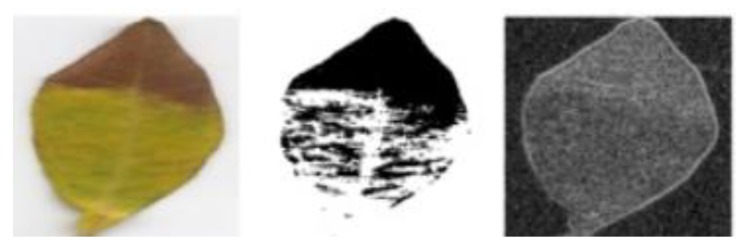
Segmentation and edge map for olive leaf disease detection [65].

For the practical experimentation of detection of plants’ diseases, an actual/real background/environment should be considered in order to evaluate the performance of the DL model more accurately. In most of the above approaches, the selected datasets considered plain backgrounds which are not realistic scenarios for identification and classification of the diseases [25,27,32,56,57,58,60,61,65,72,77,78], except for a few of them that have considered the original backgrounds [35,59,68,70,73,74]. The output of the visualization techniques used in several researches are shown in Figure 4, Figure 5, Figure 6, Figure 7, Figure 8, Figure 9, Figure 10 and Figure 11.

In Figure 4, feature maps from the first to the fifth hidden layer are shown as the neuron in a feature map having identical features at different positions of an image. Starting from the first layer (a), the features in feature maps represent separate pixels to normal lines, whereas the fifth layer shows some particular parts of the image (h).

Two types of visualization maps are shown in Figure 5, namely, heat map and saliency map techniques. The heat maps identify the diseases shown as red boxes in the input image, but it should be noted that one disease marked in (d) has not been detected. This problem was resolved in the saliency map technique after the application of the guided back-propagation [55]; all the spots of plant disease were successfully identified thanks to a method which is superior to the heat map. 

Figure 6 represents the heat map to detect the disease in maize plants. First, the image was represented in the form of the probability of each portion containing disease. Then, the probabilities were placed into the form of a matrix in order to denote the outcome of all the areas of the input image.

A new visualization technique was proposed in [67] as shown in Figure 8 and Figure 9. In Figure 8a, the input image was regenerated for student/teacher architecture [67], and a single channel heat map was produced after the application of simple aggregation on the channels of the regenerated image (Figure 8b). Then, a simple binary threshold algorithm was applied to obtain sharp symptoms of diseases in the plant. Then, [67] indicated the significance of the proposed technique by comparing it with the other visualization techniques as shown in Figure 9. On the left hand side, LRP-Z, LRP-Epsilon, and gradient did not identify plant diseases clearly. However, the Deep Taylor approach produced better results but indicated some portion of the leaf disease. On the right hand side, an imperfect localization of the plant disease was shown in grad-cam techniques which was resolved in the proposed technique by the use of a decoder [67].

In order to find the significance of CNN architectures to differentiate between various diseases of plants, the feature maps were obtained as shown in Figure 10. The result proves a good performance of the proposed CNN model as it clearly identifies the disease in plants [85]. 

In Figure 11 the segmentation and edged maps were obtained to identify the diseases in plants. It is noted that the yellow colored area is marked as white surface in the segmentation map to show the affected part of the leaf.

### 2.2. New/Modified DL Architectures for Plant-Disease Detection

According to some of the research papers, new/modified DL architectures have been introduced to obtain better/transparent detection of plant disease, such as [86] presented improved GoogLeNet and Cifar-10 models and their performance compared with AlexNet and VGG. It was found that improved versions of these state-of-the-art models produced a remarkable accuracy of 98.9%. In [87], a new DL model was introduced to obtain more accurate detection of plant diseases as compared to SVM, AlexNet, GoogLeNet, ResNet-20, and VGG-16 models. This model achieved 97.62% accuracy for classifying apple plant diseases. Moreover, the dataset extended in 13 different ways (rotation of 90°, 180°, 270° and mirror symmetry (horizontal symmetry), change in contrast, sharpness and brightness). Moreover, the whole dataset was transformed into Gaussian noise and PCA jittering as well. Furthermore, the selection of dataset was explained by the help of plots to prove the significance of extending the dataset. A new CNN model named LeafNet was introduced in [88] to classify the tea leaf diseases and achieved higher accuracy than Support Vector Machine (SVM) and Multi-Layer Perceptron (MLP). In [89], two DL models named modified MobileNet and reduced MobileNet were introduced, and their accuracy was near to the VGG model; the reduced MobileNet actually got 98.34% classification accuracy and had a fewer number of parameters as compared to VGG which saves time in training the model. A state-of-the-art DL model was proposed in [90] named PlantdiseaseNet which was remarkably suitable for the complex environment of an agricultural field. In [85], five types of apple plant diseases were classified and detected by the state-of-the-art CNN model named VGG-inception architecture. It outclassed the performance of many DL architectures like AlexNet, GoogLeNet, several versions of ResNet, and VGG. It also presented inter object/class detection and activation visualization; it was also mentioned for its clear vision of diseases in the plants.

**Figure 12:**
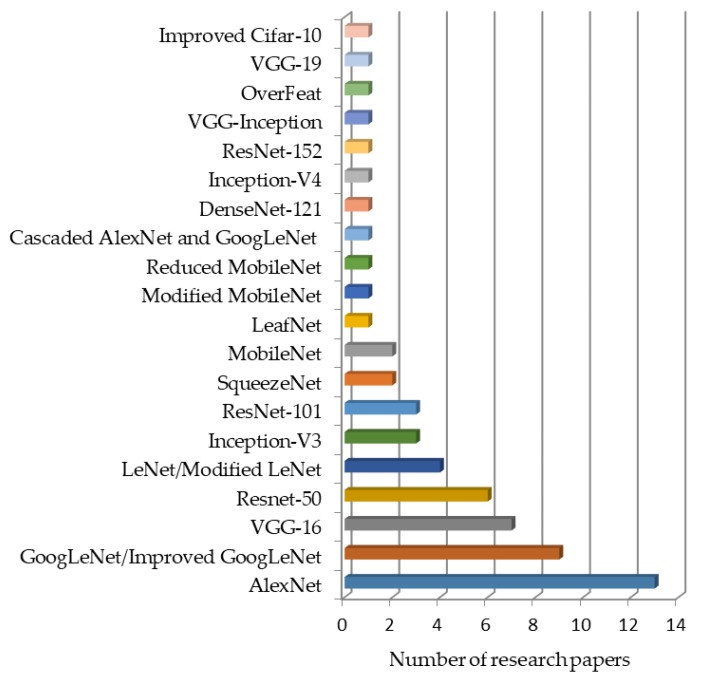
Deep learning models used in the particular number of research papers.

A bar chart presented in Figure 12 indicates, from the most to the least frequently used, DL models for plant disease detection and classification. It can be clearly seen that the AlexNet model has been used in most of the researches. GoogLeNet, VGG-16, and ResNet-50 are the next most commonly used DL models. Similarly, there are some improved/cascaded versions (Improved Cifar-10, VGG-Inception, Cascaded AlexNet with GoogLeNet, reduced/modified MobileNet, modified LeNet, and modified GoogLeNet), which have been used for plant disease identification. 

Summing up Section 2, all the DL approaches along with the selected plant species and performance metrics are shown in Table 3.

## 3. Hyper-Spectral Imaging with DL Models

For early detection of plant diseases, several imaging techniques like multispectral imaging [91], thermal imaging, fluorescence and hyperspectral imaging are used [92]. Among them, hyperspectral imaging (HSI) is the focus of recent research. For example, [93] used hyperspectral imaging (HSI) to detect tomato plant diseases by identifying the region of interest, and a feature ranking-KNN (FR-KNN) model produced a satisfactory result for the detection of diseased and healthy plants. In the recent approach, HSI was used for the detection of an apple disease. Moreover, the redundancy issue was resolved by an unsupervised feature selection procedure known as Orthogonal Subspace Projection [94]. In [95], leaf diseases on peanuts were detected by HSI by identifying sensitive bands and hyperspectral vegetation index. The tomato disease detection was done by SVM classifiers based on HSI, and their performance was evaluated by F1-score, accuracy, specificity, and sensitivity [96].

Recently, HSI has been used with machine learning (ML) for the detection of plant diseases. For example, [97] described ML techniques for hyperspectral imaging for many agricultural applications. Moreover, ML with HSI have been used for three ML models, implemented by using hyperspectral measurement technique for the detection of leaf rust disease [98]. For wheat disease detection, [99] used Random Forest (RF) classifier with multispectral imaging technique and achieved accuracy of 89.3%. Plants’ diseases were also detected by SVM based on hyperspectral data and achieved accuracy of more than 86% [100]. There are some other ML approaches based on HSI [101], but this review is focused on DL approaches based on HSI, presented below.

**Figure 13:**
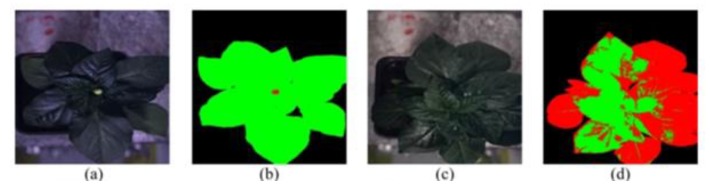
Sample images of OR-AC-GAN (a hyperspectral imaging model) [112].

**Figure 14:**
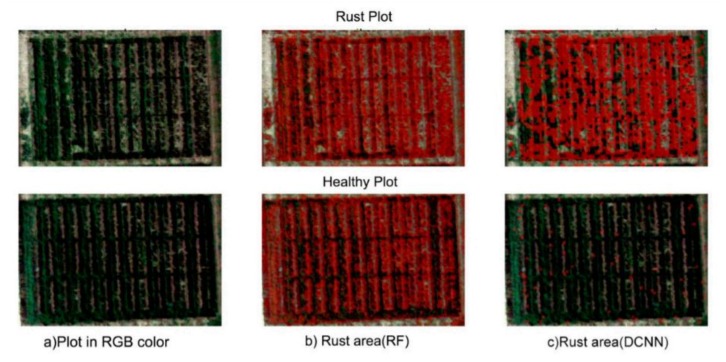
Hyperspectral images by UAV: (**a**) RGB color plots, (**b**) Random-Forest classifier, and (**c**) proposed multiple Inception-ResNet model [114].

The DL has been used to classify the hyperspectral images for many applications. For medical purposes, this technology is very useful as it is used for the classification of head/neck cancer in [102]. In [103], a DL approach based on HSI was proposed through contextual information as it provides spectral and spatial features. A new 3D-CNN architecture allowed for a fast, accurate, and efficient approach to classify the hyperspectral images in [104]. This architecture not only used the spectral information (as used in previous CNN techniques [105]) but also ensured that the spatial information was also taken into account. In [106], the feature extraction procedure was used with CNN for hyperspectral image classification and used dropout and L2 regularization methods in order to prevent overfitting. Just like CNN models used for hyperspectral imaging classification, RNN models are also used with HSI as described in [107,108]. In the domain of plant disease detection, some researches utilized Hyperspectral Imaging (HSI) along with DL models to observe clearer vision for symptoms of plant diseases. A hybrid method to classify the hyperspectral images was proposed in [109] consisting of DCNN, LR, and PCA and got better results compared to the previous methods for classification tasks. In [110], a detailed review of DL with HSI technique was provided. In order to avoid the overfitting and improve accuracy, a detailed comparison provided between several DL models like 1D/2D-CNN (2D-CNN better result), LSTM/GRU (both faced overfitting), 2D-CNN-LSTM/GRU (still overfitting) was observed. Therefore, a new hybrid approach from Convolutional and Bidirectional Gated Recurrent Network named 2D-CNN-BidLSTM/GRU was proposed for the hyperspectral images, which resolved the problem of overfitting and achieved 0.75 F1-score and 0.73 accuracy for wheat diseases detection [111]. According to [112], a hyperspectral proximal-sensing procedure based on the newest DL technique named Generative Adversarial Nets (GAN) was proposed in order to detect tomato plant disease before its clear symptoms appeared (as shown in Figure 13). In [84], a 3D-CNN approach was proposed for hyperspectral images to identify the Charcoal rot disease in soybeans and the CNN model was evaluated by accuracy (95.76%) and F1-score (0.87). The saliency map visualization was used, and the most delicate wavelength resulted as 733 nm, which approximately lies in the region of the wavelength of NIR. For the detection of potato virus, [113] described it by DL on the hyperspectral images and achieved acceptable values of precision (0.78) and recall (0.88). In [114], a DL model named multiple Inception-Resnet model was developed by using both spatial and spectral data on hyperspectral UAV images to detect the yellow rust in wheat (as shown in Figure 14). This model achieved an 85% accuracy, which is quite a lot higher than the RF-classifier (77%). 

From this section, we can conclude that, although there are some DL models/architectures developed for hyperspectral image classification in the application of plant disease detection, this is still a fertile area of research and should lead to improvements for better detection of plants’ diseases [115] in different situations, like various conditions of illumination, considering real background, etc. 

In Figure 13, the resultant images are taken from the proposed method described in [112]. The green-colored portion indicates the healthy part of the plant; the red portion denotes the infected portion. Note that (**a**) and (**b**) are the healthy plant images as there is no red color indication, whereas (**c**) has infected disease which can be seen in its corresponding figure (**d**).

A comparison of proposed DCNN with RF classifier and RGB colored hyperspectral images are shown in Figure 14. The red color label indicates the portion infected by rust. It should be observed that the rust plots were identified in an almost similar manner (see (b) and (c) of first row), but in the healthy plot, there was a large portion covered by the red label in (b) as compared to (c), which shows a wrong classification by RF model [114].

## 4. Conclusions and Future Directions

This review explained DL approaches for the detection of plant diseases. Moreover, many visualization techniques/mappings were summarized to recognize the symptoms of diseases. Although much significant progress was observed during the last three to four years, there are still some research gaps which are described below:

- In most of the researches (as described in the previous sections), the PlantVillage dataset was used to evaluate the accuracy and performance of the respective DL models/architectures. Although this dataset has a lot of images of several plant species with their diseases, it has a simple/plain background. However, for a practical scenario, the real environment should be considered.
- Hyperspectral/multispectral imaging is an emerging technology and has been used in many areas of research (as described in Section 3). Therefore, it should be used with the efficient DL architectures to detect the plants’ diseases even before their symptoms are clearly apparent.
- A more efficient way of visualizing the spots of disease in plants should be introduced as it will save costs by avoiding the unnecessary application of fungicide/pesticide/herbicide.
- The severity of plant diseases changes with the passage of time, therefore, DL models should be improved/modified to enable them to detect and classify diseases during their complete cycle of occurrence.
- DL model/architecture should be efficient for many illumination conditions, so the datasets should not only indicate the real environment but also contain images taken in different field scenarios.
- A comprehensive study is required to understand the factors affecting the detection of plant diseases, like the classes and size of datasets, learning rate, illumination, and the like.

## Figures and Tables

**Table 1:** Comparison of state-of-the-art deep learning models.

Deep Learning Models	Parameters	Key Features and Pros/Cons
LeNet	60k	First CNN model. Few parameters as compared to other CNNmodels. Limited capability of computation
AlexNet	60M	Known as the first modern CNN. Best image recognition performance at its time. Used ReLU to achieve better performance. Dropout technique was used to avoid overfitting
OverFeat	145M	First model used for detection, localization, and classification of objects through a single CNN. Large number of parameters as compared to AlexNet
ZFNet	42.6M	Reduced weights (as compared to AlexNet) by considering 7 × 7 kernels and improved accuracy
VGG	133M–144M	3 × 3 receptive fields were considered to include more number of non-linearity functions which made decision function discriminative. Computationally expensive model due to large number of parameters
GoogLeNet	7M	Fewer number of parameters as compared to AlexNet model. Better accuracy at its time
ResNet	25.5M	Vanishing gradient problem was addressed. Better accuracy than VGG and GoogLeNet models
DenseNet	7.1M	Dense connections between the layers. Reduced number of parameters with better accuracy
SqueezeNet	1.25M	Similar accuracy as AlexNet with 50 times lesser parameters. Considered 1 × 1 filters instead of 3 × 3 filters. Input channels were decreased. Large activation maps of convolution layers
Xception	22.8M	A depth-wise separable convolution approach. Performed better than VGG, ResNet, and Inception-v3 models
MobileNet	4.2M	Considered the depth-wise separable convolution concept. Reduced parameters significantly. Achieved accuracy near to VGG and GoogLeNet
Modified/Reduced MobileNet	0.5/0.54M	Lesser number of parameters as compared to MobileNet. Similar accuracy as compared to MobileNet
VGG-Inception	132M	A cascaded version of VGG and inception module. The number of parameters were reduced by substituting 5 × 5 convolution layers with two 3 × 3 layers. Testing accuracy was increased as compared to many well-known DL models like AlexNet, GoogLeNet, Inception-v3, ResNet, and VGG-16.

**Table 2:** Visualization mapping/techniques used in several approaches.

Visualization Techniques/Mappings	References
Visualization of features having filter from first to final layer	[27]
Visualize activations in first convolutional layer	[25]
Saliency map visualization	[55]
Classification and localization of diseases by bounding boxes	[68]
Heat maps were used to identify the spots of the disease	[70]
Feature map for the diseased rice plant	[78]
Symptoms visualization method	[72]
Feature and spatial core maps	[73]
Color space into HSV and K-means clustering	[74]
Feature map for spotting the diseases	[77]
Image segmentation method	[66]
Reconstruction of images on discriminant regions, segmentation of images by binary threshold theorem, and heat map construction	[67]
Saliency map visualization	[84]
Saliency map, 2D and 3D contour, mesh graph image	[82]
Activation visualization	[85]
Segmentation map and edge map	[65]

**Table 3:** Comparison of several DL approaches in terms of various performance metrics.

DL Architectures/Algorithms	Datasets	Selected Plant/s	Performance Metrics (and Their Results)	Refs
CNN	PlantVillage	Maize	CA (92.85%)	[56]
AlexNet, GoogLeNet, ResNet	PlantVillage	Tomato	CA by ResNet which gave the best value (97.28%)	[57]
LeNet	PlantVillage	Banana	CA (98.61%), F1 (98.64%)	[32]
AlexNet, ALexNetOWTBn, GoogLeNet, Overfeat, VGG	PlantVillage and in-field images	Apple, blueberry, banana, cabbage, cassava, cantaloupe, celery, cherry, cucumber, corn, eggplant, gourd, grape, orange, onion	Success rate of VGG (99.53%) which is the best among all	[58]
AlexNet, VGG16, VGG 19, SqueezeNet, GoogLeNet, Inceptionv3, InceptionResNetv2, ResNet50, Resnet101	Real field dataset	Apricot, Walnut, Peach, Cherry	F1(97.14), Accuracy (97.86 ± 1.56) of ResNet	[35]
Inceptionv3	Experimental field dataset	Cassava	CA (93%)	[59]
CNN	Images taken from the research center	Cucumber	CA (82.3%)	[60]
Super-Resolution Convolutional Neural Network (SCRNN)	PlantVillage	Tomato	Accuracy (~90%)	[61]
CaffeNet	Downloaded from the internet	Pear, cherry, peach, apple, grapevine	Precision (96.3%)	[27]
AlexNet and GoogLeNet	PlantVillage	Apple, blueberry, bell pepper, cherry, corn, peach, grape, raspberry, potato, squash, soybean, strawberry, tomato	CA (99.35%) of GoogLeNet	[25]
AlexNet, GoogLeNet, VGG- 16, ResNet-50,101, ResNetXt-101, Faster RCNN, SSD, R-FCN, ZFNet	Image taken in real fields	Tomato	Precision (85.98%) of ResNet-50 with Region based Fully Convolutional Network(R-FCN)	[68]
CNN	Bisque platform of Cy Verse	Maize	Accuracy (96.7%)	[70]
DCNN	Images were taken in real field	Rice	Accuracy (95.48%)	[78]
AlexNet, GoogLeNet	PlantVillage	Tomato	Accuracy (0.9918 ± 0.169) of GoogLeNet	[72]
VGG-FCN-VD16 and VGG-FCN-S	Wheat Disease Database 2017	Wheat	Accuracy (97.95%) of VGG-FCN-VD16	[73]
VGG-A, CNN	Images were taken in real field	Radish	Accuracy (93.3%)	[74]
AlexNet	Images were taken in real field	Soybean	CA (94.13%)	[77]
AlexNet and SqueezeNet v1.1	PlantVillage	Tomato	CA (95.65%) of AlexNet	[62]
DCNN, Random forest, Support Vector Machine and AlexNet	PlantVillage dataset, Forestry Image dataset and agricultural field in China	Cucumber	CA (93.4%) of DCNN	[66]
Teacher/student architecture	PlantVillage	Apple, bell pepper, blueberry, cherry, corn, orange, grape, potato, raspberry, peach, soybean, strawberry, tomato, squash	Training accuracy and loss (~99%,~0–0.5%), validation accuracy and loss (~95%, ~10%)	[67]
Improved GoogLeNet, Cifar-10	PlantVillage and various websites	Maize	Top-1 accuracy (98.9%) of improved GoogLeNet	[86]
MobileNet, Modified MobileNet, Reduced MobileNet	PlantVillage dataset	24 types of plant	CA (98.34%) of reduced MobileNet	[89]
VGG-16, ResNet-50,101,152, Inception-V4 and DenseNets-121	PlantVillage	Apple, bell pepper, blueberry, cherry, corn, orange, grape, potato, raspberry, peach, soybean, strawberry, tomato, squash	Testing accuracy (99.75%) of DenseNets	[63]
User defined CNN, SVM, AlexNet, GoogLeNet, ResNet-20 and VGG-16	Images were taken in real field	Apple	CA (97.62%) of proposed CNN	[87]
AlexNet and VGG-16	PlantVillage	Tomato	CA (AlexNet)	[64]
LeafNet, SVM, MLP	Images were taken in real field	Tea leaf	CA (90.16%) of LeafNet	[88]
2D-CNN-BidGRU	Real wheat field	wheat	F1 (0.75) and accuracy (0.743)	[111]
OR-AC-GAN	Real environment	Tomato	Accuracy (96.25%)	[112]
3D CNN	Real environment	Soybean	CA (95.73%), F1-score (0.87)	[84]
DCNN	Real environment	Wheat	Accuracy (85%)	[114]
ResNet-50	Real environment	Wheat	Balanced Accuracy (87%)	[79]
GPDCNN	Real environment	Cucumber	CA (94.65%)	[81]
VGG-16, AlexNet	PlantVillage, CASC-IFW	Apple, banana	CA (98.6%)	[82]
LeNet	Real environment	Grapes	CA (95.8%)	[83]
PlantDiseaseNet	Real environment	Apple, bell-pepper, cherry, grapes, onion, peach, potato, plum, strawberry, sugar-beets, tomato, wheat	CA (93.67%)	[90]
LeNet	PlantVillage	Soybean	CA (99.32%)	[71]
VGG-Inception	Real environment	Apple	Mean average accuracy (78.8%)	[85]
Resnet-50, Inception-V2, MobileNet-V1	Real environment	Banana	Mean average accuracy (99%) of ResNet-50	[69]
Modified LeNet	PlantVillage	Olives	True positive rate (98.6 ± 1.47%)	[65]

## Abbreviations

The abbreviations used in this manuscript are given as under:

ML	Machine Learning
DL	Deep Learning
CNN	Convolutional Neural network
DCNN	Deep Convolutional Neural Network
ILSVRC	ImageNet Large Scale Visual Recognition Challenge
RF	Random Forest
CA	Classification Accuracy
LSTM	Long Short-Term Memory
IoU	Intersection of Union
NiN	Network in Network
RCN	Region based Convolutional Neural Network
FCN	Fully Convolutional Neural Network
YOLO	You Only Look Once
SSD	Single Shot Detector
PSPNet	Pyramid Scene Parsing Network
IRRCNN	Inception Recurrent Residual Convolutional Neural Network
IRCNN	Inception Recurrent Convolutional Neural Network
DCRN	Densely Connected Recurrent Convolutional Network
INAR-SSD	Single Shot Detector with Inception module and Rainbow concatenation
R2U-Net	Recurrent Residual Convolutional Neural Network based on U-Net model
SVM	Support Vector Machines
ELM	Extreme Learning Machine
KNN	K-Nearest Neighbor
SRCNN	Super-Resolution Convolutional Neural Network
R-FCN	Region-based Fully Convolutional Networks
ROC	Receiver Operating Characteristic
PCA	Principal Component Analysis
MLP	Multi-Layer Perceptron
LRP	Layer-wise Relevance Propagation
HSI	Hyperspectral Imaging
FRKNN	Feature Ranking K-Nearest Neighbor
RNN	Recurrent Neural Network
ToF	Time-of-Flight
LR	Logistic Regression
GRU	Gated Recurrent Unit
AN	Generative Adversarial Nets
GPDCNN	Global Pooling Dilated Convolutional Neural Network
2D-CNN-BidGRU	2D-Convolutional-Bidirectional Gated Recurrent Unit Neural Network
OR-AC-GAN	Outlier Removal-Auxiliary Classifier-Generative Adversarial Nets
